# Unfractionated bone marrow cells attenuate paraquat-induced glomerular injury and acute renal failure by modulating the inflammatory response

**DOI:** 10.1038/srep23287

**Published:** 2016-03-18

**Authors:** Sing-Yi Gu, Ti-Yen Yeh, Shih-Yi Lin, Fu-Chuo Peng

**Affiliations:** 1 Graduate Institute of Toxicology, College of Medicine, National Taiwan University, Taipei, Taiwan

## Abstract

The aim of this study was to evaluate the efficacy of unfractionated bone marrow cells (BMCs) in attenuating acute kidney injury (AKI) induced by paraquat (PQ) in a mouse model. PQ (55 mg/kg BW) was intraperitoneally injected into C57BL/6 female mice to induce AKI, including renal function failure, glomerular damage and renal tubule injury. Glomerular podocytes were the first target damaged by PQ, which led to glomerular injury. Upon immunofluorescence staining, podocytes depletion was validated and accompanied by increased urinary podocin levels, measured on days 1 and 6. A total of 5.4 × 10^6^ BMCs obtained from the same strain of male mice were injected into AKI mice through the tail vein at 3, 24, and 48 hours after PQ administration. As a result, renal function increased, tubular and glomerular injury were ameliorated, podocytes loss improved, and recipient mortality decreased. In addition, BMCs co-treatment decreased the extent of neutrophil infiltration and modulated the inflammatory response by shifting from pro-inflammatory Th1 to an anti-inflammatory Th2 profile, where IL-1β, TNF-α, IL-6 and IFN-γ levels declined and IL-10 and IL-4 levels increased. The present study provides a platform to investigate PQ-induced AKI and repeated BMCs injection represents an efficient therapeutic strategy.

Paraquat (1,1′-dimethyl-4,4′-bipyridylium dichloride; PQ) is a non-selective herbicide, defoliant, and desiccant that has been widely and commonly used worldwide since the 1960 s^1^,^2^. Most cases of PQ intoxication result from suicide attempts or accidental exposure^3^. Following ingestion, PQ is rapidly distributed to various tissues, especially the kidney, lung and liver^4^. At 3 hours after PQ exposure, the concentration is highest in the kidney and leads to acute kidney injury (AKI), which magnifies the toxicity in the lung and other organs^5^,^6^. In PQ-induced AKI, patients usually develop tubular degeneration and formation of granular eosinophilic cytoplasm in the proximal and distal convoluted tubules^5^. In clinical practice, immunosuppressive therapy including methylprednisolone (MP), cyclophosphamide (CP), and dexamethasone (DEX) has been prescribed for the treatment of PQ poisoning, although the mortality rate remains high^7^,^8^. PQ is not removed by dialysis, and hemodialysis is used only as a supportive treatment for patients who develop kidney failure^9^. Thus, maintaining renal function in patients suffering from PQ poisoning remains a therapeutically important treatment strategy^10^,^11^. However, there is no specific antidote or effective therapy for PQ poisoning; therefore, effective therapies are urgently needed.

The mechanism of PQ toxicity involves a series of cyclic reduction-oxidation reactions, with sequential depletion of reduced nicotinamide adenine dinucleotide phosphate (NADPH) and generation of PQ radicals and reactive oxygen species (ROS)^12^,^13^. The following pathological changes can be observed after PQ exposure: (1) the renal tubules lose their characteristic appearance, and their lining epithelial cells develop cytoplasmic vacuolation; (2) the glomerulus becomes degenerated, and the renal blood vessels become congested; and (3) the inter-tubular spaces are infiltrated by inflammatory leukocytes^14^. However, the specific mechanisms of PQ-induced AKI have not been fully elucidated.

The concept of bone marrow-derived cells engraftment involves the differentiation of these cells into functional somatic cells capable of repairing injured tissue. Additionally, bone marrow-derived cells transplantation may support the healing of damaged tissues by exerting potent immunosuppressive effects and secreting soluble factors that regulate the pro-inflammatory cascade to promote tissue remodeling and cellular regeneration^15^. Many studies have shown that bone marrow-derived cells may be involved in tissue turnover and regeneration, including in the kidney. In particular, unfractionated bone marrow cells (BMCs) have been shown to regenerate tissue after cisplatin-induced acute tubular injury, improve renal function and ameliorate inflammation^16^. The selective recruitment and localization of bone marrow-derived cells to the kidney vasculature result in structural and functional recovery, as well as increased survival^17^. Bone marrow cells are incorporated into the glomerulus during recovery from experimentally induced glomerulonephritis^18^, and bone marrow-derived cells transplantation led to the formation of bone marrow-derived podocytes and mesangial cells in a mouse model of Alport’s syndrome, a hereditary nephropathy^19^. Given the poor prognosis of AKI, the promise of bone marrow-derived cells transplantation therapy to aid regeneration and organ recovery is an attractive therapeutic strategy.

Oxidative stress and renal tubule injury are important factors in AKI caused by PQ poisoning. Currently, anti-oxidative stress therapy and glucocorticoid treatments are clinically applied. However, the survival rates of patients with PQ poisoning still remain as low. Novel insights into the toxicity mechanisms and potential therapeutic strategy developments are promising to reduce the high mortality of PQ poisoning. In the present study, we first attempted to characterize the mechanisms of PQ-induced toxicity in C57BL/6 mice, including the survival rate, degree of renal function, and pathological alterations. Furthermore, we sought to evaluate whether BMCs were capable of minimizing or preventing kidney injuries induced by PQ. Inflammatory cytokine and chemokine analysis was performed to assess the potential of BMCs transplantation in reducing PQ-induced nephrotoxicity.

## Results

### BMCs improved survival in PQ-treated mice

**Figure 1:**
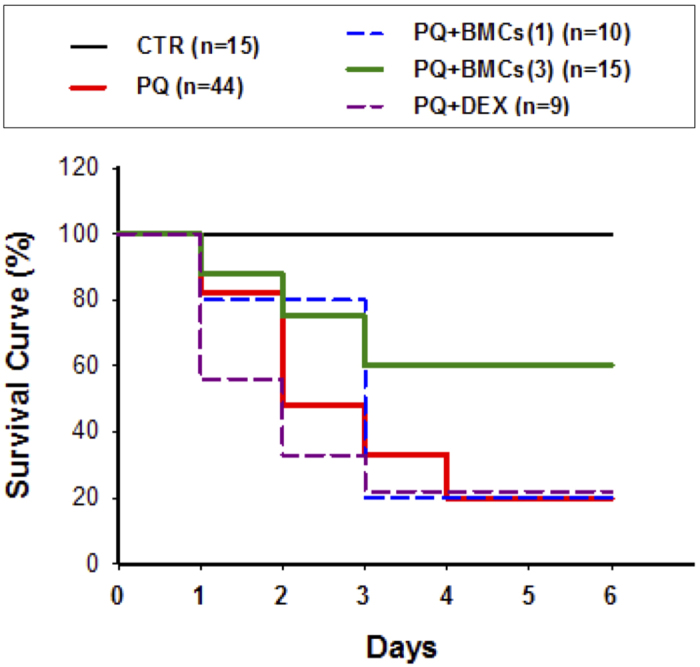
The survival rate of C57BL/6 mice in the various treatment groups. The treatment groups included the control group (CTR, normal saline), PQ group (55 mg/kg BW), PQ + BMCs (1) (single BMCs co-treatment with PQ), PQ + BMCs (3) group (triple BMCs co-treatment with PQ) and PQ + DEX group (10 mg/kg BW DEX co-treatment with PQ). Survival rate was recorded from day 1 to day 6 after PQ treatment.

PQ exposure is a risk factor for the development of nephrotoxicity. Our experiments first evaluated and determined the dose of PQ that would cause AKI following intraperitoneal (ip) administration in C57BL/6 female mice. Based on previous reports and to simulate the clinical phenomenon of PQ poisoning, PQ was administered ip at doses of 55, 65, and 70 mg/kg BW to evaluate acute toxicity in C57BL/6 female mice. The survival rate was recorded on days 1–6 following administration of PQ. The survival rates of C57BL/6 female mice treated with 55, 60 and 70 mg/kg BW of PQ were 20, 10, and 0%, respectively, on day 6 (data not shown). These results were similar to the mortality rate of clinical patients with PQ-induced nephrotoxicity^20^. After that PQ treatment at a dose of 55 mg/kg BW, the survival rates of mice that also received BMCs were evaluated from day 1–6. BMCs were identified using flow cytometry analysis showed that most of the BMCs were positive for mesenchymal markers CD105, CD29, and CD90 and negative for markers of hematopoietic lineages CD31, CD34, and CD45 (Supplementary Fig. 1A). After 24 h of adherent growth, the BMCs morphologically resembled fibroblasts (Supplementary Fig. S1B). The treatment process included single or triple BMCs co-treatment, and the detailed protocol is described in the Material and Methods section. Transplanted BMCs being detected on day 3 in PQ + BMCs (3) mice by using PKH26 pre-labeled BMCs in kidney section (Supplementary Fig. S2). After 6 days, the survival rate of PQ-treated animals was 20%, whereas normal saline treatment in the control group had no adverse effect on survival. In the single BMCs co-treatment group (PQ + BMCs (1)), the survival rate was also 20%. However, the triple BMCs co-treatment group (PQ + BMCs (3)) showed a significantly improved survival rate of 60%. Additionally, DEX, an anti-inflammatory steroid drug, did not improve the survival rate of PQ-treated animals (22%) (PQ + DEX) (Fig. 1). This result showed that anti-inflammatory drug therapy with DEX did not efficiently improve survival following PQ poisoning, whereas repeated BMCs co-treatment exhibited a positive effect on survival.

### Triple BMCs co-treatment improved the biochemical profiles of PQ-induced mice

To study the therapeutic effects of BMCs in PQ-induced AKI, renal function was analyzed in PQ-only and triple BMCs co-treated mice. Serum blood urea nitrogen (BUN) and Creatinine (CRE) levels were significantly elevated compared to the control group after PQ administration, with a peak at day 1; the serum BUN level was 145.81 ± 8.66 mg/dl (*p *< 0.001), and the CRE level was 0.84 ± 0.47 mg/dl (*p *< 0.001). Cell therapy with triple BMCs injection significantly decreased the serum BUN and CRE levels compared with the PQ-only group (Table 1).

The urine protein and CRE ratio (uPCR) was significantly higher in PQ-only mice; this ratio reached its highest level of 15.18 ± 4.15 (*p *< 0.001) following PQ treatment on day 1. Additionally, in triple BMCs co-treated mice, the uPCR was significantly lower than in the PQ-only group and was similar to that in control mice (Table 1). These results showed that triple BMCs co-treatment markedly improved renal function by lowering serum BUN and CRE levels and reducing the uPCR.

### Effect of triple BMCs co-treatment on PQ-induced glomerular and tubular injury

**Figure 2:**
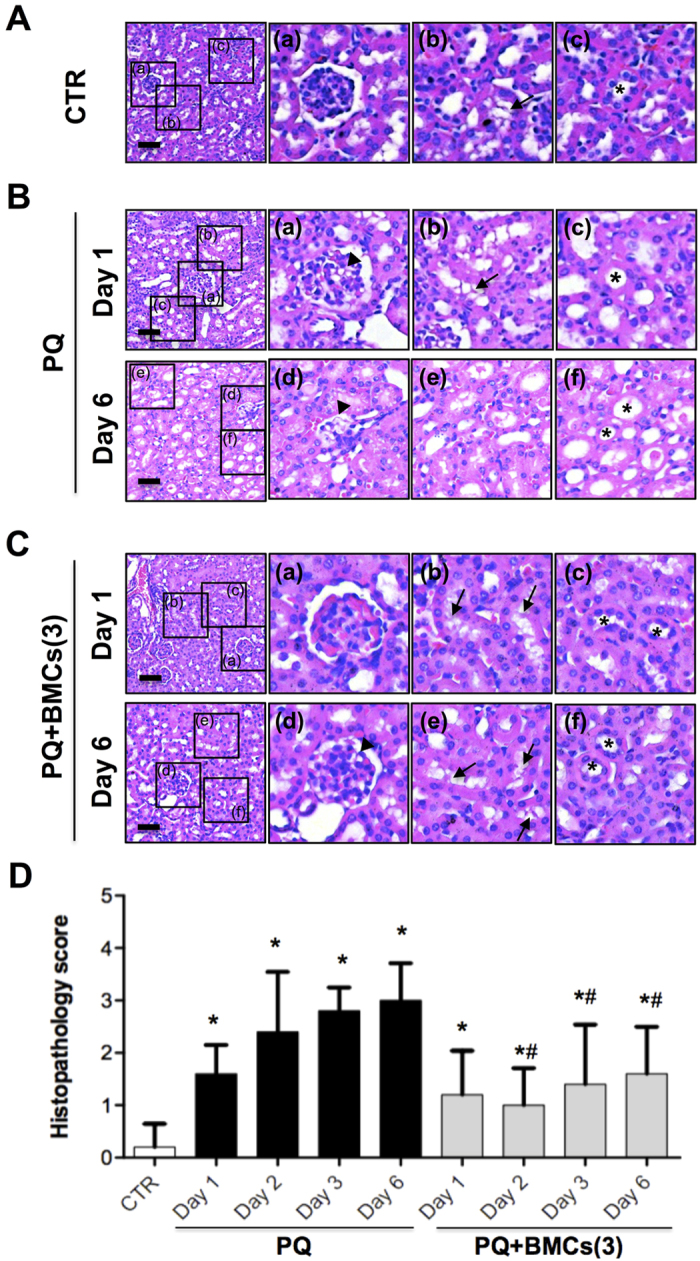
Histopathological examination of kidney tissue by HE staining. (**A**) The control group displayed standard kidney histopathology. a, Complete Bowman’s capsule and glomerulus architecture. b, Renal tubule with distributed brush border (arrow). c, Regular nuclear arrangement in renal tubule (asterisk). (**B**) The PQ-only group displayed histopathological changes. On day 1, kidneys showed mild changes: a, incomplete Bowman’s capsule with vacuolization in the glomerulus (arrowhead); b, mild loss of brush border in the renal tubule; and c, loss of polarity of tubular cells. On day 6, kidneys showed severe histopathological change: d, glomerular shrinkage and decreased space between the glomerulus and Bowman’s capsule (arrowhead); e, loss of brush border in the renal tubule, along with cytoplasmic flattening and loss of the epithelial cells; and f, loss of polarity of tubular cells and degeneration in the renal tubule (asterisk). (**C**) Triple BMCs administration improved kidney histopathology following PQ treatment. a and d, Complete Bowman’s capsule and glomerulus architecture on day 1 and mild vacuolization in the glomerulus on day 6 (arrowhead). b and e, Renal tubule with distributed brush border (arrows). c and f, Regular nuclear arrangement, and well-development renal tubule (asterisk). Scale bar is 50 μm. (**D**) Histopathological examination of kidney tissue of PQ administrated mice. Histopathology scoring of renal glomerular degeneration and renal tubule necrosis by a veterinary pathologist: grade 0: no injury; grade 1: minimal injury with less than 10% of cells exhibiting degeneration or necrosis; grade 2: mild injury involving 10–25% of cells; grade 3: moderate injury involving 25–40% of cells; grade 4: marked injury involving 40–50% of cells = grade 4; grade 5: severe injury involving greater than 50% of cells. N = 5, *p < 0.05 vs. control group and ^#^p < 0.05 vs. PQ-only group.

Histopathological examination was performed on days 1 and 6 following PQ-only administration, triple BMCs co-treatment and control groups. Kidney sections of control group displayed standard kidney histopathology, such as complete Bowman’s capsule, well-organized glomerulus, renal tubule with distributed brush border and regular nuclear arrangement (Fig. 2A). Kidney sections of PQ-only administration displayed severe histopathological alterations. In the glomerulus, PQ administration induced Bowman’s urinary space decrease, vacuolization in the glomerulus and glomerulus atrophy on days 1 and 6. In the renal tubule, PQ-induced mice displayed severe loss of the brush border, depletion of tubular polarity, and loss of cell nuclei in proximal convoluted tubules on day 6 (Fig. 2B). However, triple BMCs co-treatment markedly attenuated glomerular and tubular injuries by maintaining glomerulus architecture and retaining brush border polarity in renal tubule (Fig. 2C). Quantification of histopathological alterations of PQ administered mice were significantly increased and triple BMCs co-treatment markedly ameliorated histopathological score (Fig. 2D). These results indicated that PQ-induced kidney damage targeted both the glomerulus and renal tubule, although triple BMCs co-treatment successfully attenuated PQ-induced kidney injury.

### Triple BMCs co-treatment resumed podocytes damage induced by PQ administration

**Figure 3:**
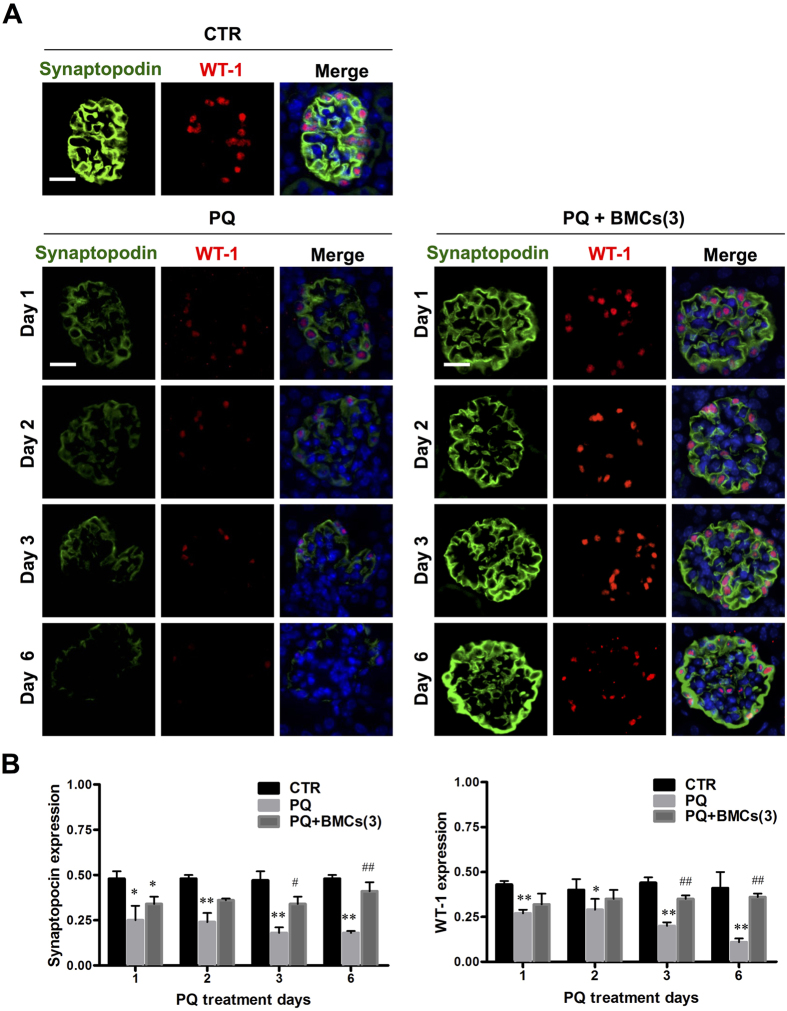
Podocytes depletion of C57BL/6 mice in the treatment groups. (**A**) Kidney tissue from mice in the control, PQ-only and PQ + BMCs (3) groups were collected on days 1, 2, 3 and 6. Kidney sections underwent double immunofluorescence staining to validate synaptopodin and WT-1 expression in the glomerulus. Synaptopodin is labeled in green (Alex488nm fluorescence) and distributed within the cytoplasm of podocytes. WT-1, a podocytes nuclear marker, is shown in red (Cy3 fluorescence). DAPI was used to stain the nucleus. These images are representative of each group. The control group displayed standard synaptopodin and WT-1 distribution. Following PQ-only treatment, synaptopodin and WT-1 expression dramatically diminished, whereas triple BMCs co-treatment resulted in markedly recovered synaptopodin and WT-1 expression. Scale bar is 50 μm, n = 6. (**B**) Quantification of glomerular synaptopodin and WT-1 expression in kidneys of the control, PQ-only and PQ + BMCs (3) groups. *p < 0.05, **p < 0.01 vs. control group. ^#^*p* < 0.05, ^##^*p* < 0.01 vs. PQ-only group.

**Figure 4:**
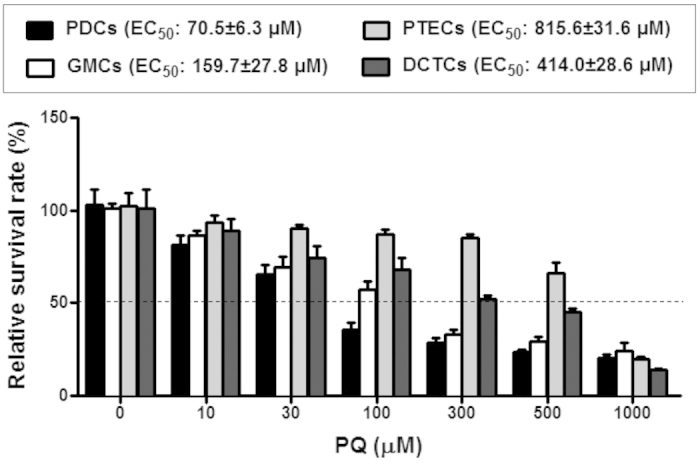
*In vitro* cytotoxicity of PQ on viability of the glomerulus and renal tubular epithelial cells. Effects of PQ on cell viability of glomerulus podocytes (PDCs), glomerulus mesangial cells (GMCs), proximal tubule epithelial cells (PTECs), and distal convoluted tubule epithelial cells (DCTCs) determined using the WST-1 assay. Cells were treated with PQ (10, 30, 100, 300, 500, and 1000 μM) for 24 h. Results are expressed as the percent of cell viability compared to the control cells. *p < 0.05, **p < 0.01 vs. control cells.

Glomerular injury is often characterized by the effacement of podocytes and loss of slit diaphragms^21^. Renal biopsies of patients with proteinuria and kidney disease most often are associated with immunofluorescence staining was performed for WT-1 and synaptopodin expression, and the results showed that PQ-only mice displayed severe loss of WT-1 and synaptopodin expression, whereas triple BMCs co-treatment resumed this expression level (Fig. 3A). According to fluorescence quantification, synaptopodin expression in the glomerulus was 0.48 ± 0.02 in the control group, whereas following PQ treatment, synaptopodin expression dramatically decreased to 0.10 ± 0.01 on day 6. WT-1 expression in the glomerulus was 0.41 ± 0.09 in the control group and remarkably decreased to 0.11 ± 0.02 after 6 days of PQ treatment. Triple BMCs co-treatment significantly improved WT-1 and synaptopodin expression compared to the PQ-only treatment group, whereas the levels were similar to those of the control group (Fig. 3B). *In vitro*, PQ cytotoxicity was evaluated in renal cell lines, including glomerulus podocytes (PDCs), glomerulus mesangial cells (GMCs), renal proximal tubule epithelial cells (PTECs), and renal distal convoluted tubule epithelial cells (DCTCs). Cellular viability analysis showed glomerulus podocytes was the most susceptible to PQ cytotoxicity. The EC_50_ value of PQ was 70.5 ± 6.3 μM for PDCs, which are approximately 2–12 fold lower than that of the GMCs, PTECs, and DCTCs (Fig. 4). These results showed that glomerular podocytes were the first target for PQ damage and led to glomerular injury, whereas triple BMCs co-treatment markedly attenuated this pathological damage.

**Figure 5:**
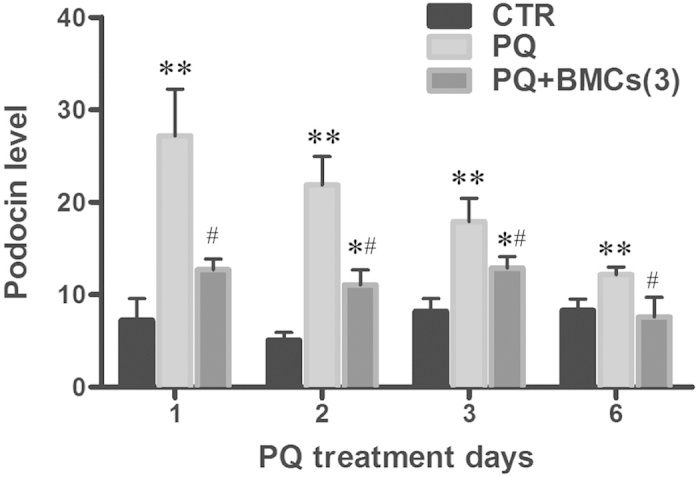
Quantification of urinary podocin in kidneys of the control, PQ-only and PQ + BMCs (3) groups. Urine samples from the control, PQ-only, and PQ + BMCs (3) groups were collected on days 1, 2, 3, and 6. Urinary podocin level examined using ELISA. The urinary podocin content was normalized to the urinary CRE concentration. All samples were assayed in triplicate. *p < 0.05, **p < 0.01 vs. control group. ^#^*p* < 0.05 vs. PQ-only group.

After glomerulus injury, podocytes can undergo depletion with the appearance in urine of structural and functional podocyte components. Monitoring podocyte loss by measuring urinary specific podocyte antigens may be promising clinically application for AKI diagnosis. The molecular component in podocyte foot processes and slit diaphragms are in the process of being understood, such as podocin, which involved in maintaining slit diaphragms^22^. In our study, urinary podocin levels were significantly increased following PQ-only treatment (the highest peak was observed at 27.19 ± 5.03 on day 1) and were markedly increased compared with the control group (7.22 ± 2.35, *p *< 0.001). In contrast, the urinary podocin level was reduced in the triple BMCs co-treatment group (12.72 ± 1.11, *p *< 0.001) at day 1 and was similar to the level observed in the control group at day 6 (7.26 ± 2.08, *p *< 0.58 relative to control group) (Fig. 5). These data show that triple BMCs co-treatment resumed podocytes loss induced by PQ administration.

### Triple BMCs co-treatment reduced neutrophil infiltration and inflammation in the kidney

Based on immunofluorescence staining, neutrophil infiltration was increased following PQ treatment but was significantly reduced by triple BMCs co-treatment. The number of Ly6G-positive neutrophils in the PQ-only group was elevated, and the highest peak was observed as 4.82 ± 0.28 cells/mm^2^ on day 3, which was significantly higher than that observed in the control group (0.84 ± 0.03/mm^2^, *p *< 0.001). In the triple BMCs co-treatment group, there were significantly fewer Ly6G-positive cells (1.17 ± 0.02/mm^2^) compared to the PQ-only group (Table 2). Thus, PQ-induced AKI was accompanied with neutrophil infiltration, and triple BMCs co-treatment showed a significant therapeutic effect against this type of inflammation.

**Figure 6:**
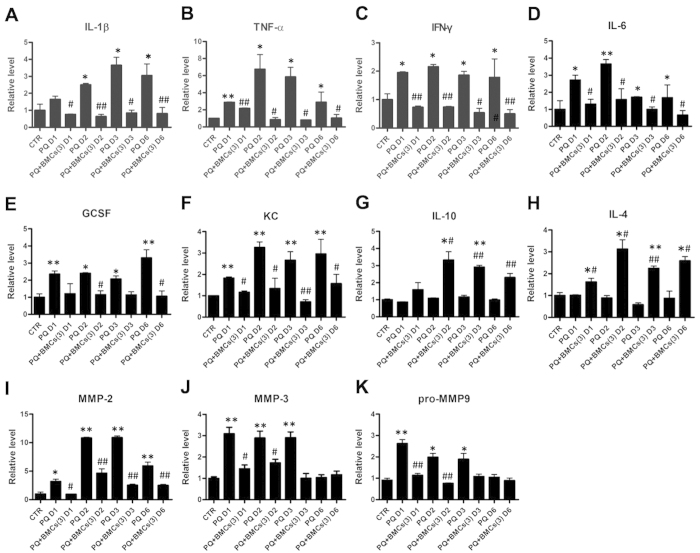
Kidney inflammatory cytokine and chemokine levels in C57BL/6 mice from the different treatment groups. Kidney supernatants from the control, PQ-only and PQ + BMCs (3) groups were collected on days 1, 2, 3 and 6. The expression levels of (**A**), IL-1β; (**B**), TNF-α; (**C**), IFN-γ; (**D**), IL-6; (**E**), GCSF; (**F**), KC; (**G**), IL-10; (**H**), IL-4; (**I**), MMP2; (**J**), MMP3; (**K**), pro-MMP9 were shown relative to the control group. *p < 0.05, **p < 0.01 vs. control group. ^#^*p* < 0.05, ^##^*p* < 0.01 vs. PQ-only group.

We further evaluated pro-inflammatory cytokines, including interleukin 1β (IL-1β), tumor necrosis factor α (TNF-α), and interferon-γ (IFN-γ) in kidney supernatants of treated animals. These results showed increased level of various cytokines in the PQ-only group compared with the control group but significantly decreased level in the triple BMCs co-treatment group (Fig. 6A–C). Moreover, as neutrophil recruitment and activation is associated with interleukin 6 (IL-6), granulocyte colony-stimulating factor (GCSF) and keratinocyte-derived chemokine (KC), we observed an increased level of these factors in the PQ-only group (Fig. 6D–F). However, triple BMCs co-treatment efficiently inhibited these factors and induced the production of IL-10 and IL-4 (Fig. 6G,H). In addition to inflammatory factors, matrix metalloproteinases (MMPs) mediate micro-vascular disruption and progression of AKI. In our results, PQ treatment resulted in significantly increased MMP2, MMP3 and pro-MMP9 levels (Fig. 6I–K), whereas levels of the endogenous inhibitors TIMP1 and TIMP2 were decreased (data not shown). Moreover, these MMP level were diminished following triple BMCs co-treatment. Together, these results showed that PQ treatment induced elevated level of numerous inflammatory factors and neutrophil infiltration, whereas triple BMCs co-treatment diminished this level of inflammation cytokines and up-regulated the anti-inflammatory factors, IL-10 and IL-4.

## Discussion

PQ poisoning is a major public health issue in many developing countries, and acute PQ poisoning leads to a high mortality rate (60–80%) within one week due to AKI^23^. In the present study, the survival rate of C57BL/6 mice that received triple BMCs co-treatment significantly increased the survival rate to 60%. In our study, PQ administration resulted in acute renal function failure, podocytes depletion, and glomerular injury, which were reversed following triple BMCs injections. Thus, the present study provides a platform to investigate PQ-induced AKI and develop efficient therapeutic strategies.

Many inflammatory mediators produced in response to PQ poisoning are secreted by neutrophils, including elastase, proteases and oxidants. Thus, immunosuppressive therapy has been used in the treatment of PQ poisoning, particularly glucocorticoids, which are widely used therapeutically to achieve immune suppression by inhibiting the NF-κB-mediated transcription of pro-inflammatory cytokines^24^. However, the utility of these immunosuppressive agents in protecting against PQ poisoning remains controversial^8^. Many clinical studies have also shown that glucocorticoid treatment did not improve the survival rate of patients with fulminant poisoning^20^. In recent years, several studies have shown that bone marrow derived cells transplantation has the ability to reduce mortality resulting from AKI^15^,^25^. In the present study, C57BL/6 mice with PQ-induced AKI, the conventional immunosuppressive drug DEX had no effect on survival (22%), whereas triple BMCs co-treatment was efficiently improved the survival rate up to 60%, although single administration had no such effect. The ability of BMCs to ameliorate AKI was closely related with the frequency and dosage of BMCs treatment, which indicates that an optimal engraftment protocol warrants further investigation.

In pathological examination, we observed the loss of polarity and brush borders in the proximal convoluted tubules, as well as glomerular injury. Many previous studies of PQ-induced nephrotoxicity have focused on the renal tubule, as kidney damage is primarily localized in the proximal tubule. However, PQ exposure has been associated with various glomerular diseases, such as glomerulonephritis^26^. Upon histology examination, glomerular injuries were observed in the PQ-only group, with incomplete Bowman’s capsules and vacuolization in the glomerulus on day 1, which further progressed to glomerular shrinkage and decreased the space between the glomerulus and Bowman’s capsule on day 6. As glomerular podocytes play a critical role in numerous glomerular diseases^21^, we were interested in whether podocytes participated in PQ-induced glomerular injury. Upon immunofluorescence staining, podocytes depletion was immediately identified after PQ treatment from day 1 until day 6, indicated by the loss of expressed synaptopodin and WT-1 protein, two specific podocyte antigens. This scenario is also supported by our *in vitro* cytotoxicity studies showing that podocytes displayed more sensitivity to PQ treatment than renal tubular epithelial cells or glomerular mesangial cells. Podocytes are highly specialized cells with important roles in maintaining the glomerular filtration barrier and producing growth factors for both mesangial and endothelial cells^22^,^27^. Synaptopodin is essential for the integrity of the podocyte actin cytoskeleton and for regulating podocyte migration. Moreover, synaptopodin serves as a marker of podocyte differentiation and maturation^28^. The WT-1 protein is highly expressed in podocytes and plays an important role in the maintenance of podocytes function^22^. Previous studies have involved immunofluorescence staining of synaptopodin and WT-1 to monitor damage to podocytes^29^,^30^. According to the above discussion results, PQ toxicity targeted and damaged podocytes, leading to glomerular injury. Podocyte loss has been proposed as a more specific marker for ongoing glomerular damage than proteinuria and albuminuria^31^. In the present study, podocyte loss paralleled the increase in urinary podocin levels, suggesting that PQ-induced AKI is closely associated with the pathological alteration of podocytes. Urinary podocin would thus be a suitable biomarker for the diagnosis and risk stratification of AKI in PQ poisoning. This present study was the first to investigate the involvement of podocytes in PQ-induced glomerular injury. The significance of these findings in relation to PQ induced podocytes damage and AKI deserves further exploration.

In AKI, pro-inflammatory cytokines and chemokines are important factors that initiate inflammation; they also play a leading role in the progression of AKI. PQ poisoning induces glomerulus injury, renal tubular epithelium damage, and leukocyte infiltration generates mediators that potentiate inflammation, including IL-1β, TNF-α, IL-6 and IFN-γ, which favor a Th1 type inflammatory response. Triple BMCs administration following PQ injury resulted in a significant decrease in pro-inflammatory cytokine levels but increased IL-10 and IL-4 level, which are associated with the Th2 anti-inflammatory response. Although they remain poorly understood, BMCs have been shown to possess immunomodulatory properties that result in the inhibition or modulation of the T cell response, and these cells also secrete various growth factors and cytokines. Previous studies have shown that mesenchymal stem cell triggering of the Th2 immune response is critical for renal regeneration following ischemic injury^32^,^33^. Our findings indicate that BMCs can modulate the inflammatory response by shifting it from a pro-inflammatory Th1 profile to an anti-inflammatory Th2 profile. Moreover, the beneficial effects of BMCs are primarily mediated via complex paracrine actions that promote a protective response important for tissue regeneration in PQ-induced AKI.

MMPs are a family of zinc-dependent proteases responsible for extracellular matrix turnover, and they are also known to modulate the tissue microenvironment^34^. Recently, MMPs, particularly MMP2 and MMP9, have been shown to play major roles in pathological lesions of various organs^35^,^36^. MMP3 is induced by pro-inflammatory cytokines such as TNF-α and IL-1β and cleaves pro-MMP9 to produce active MMP9^37^. In addition, activated MMP2 and MMP9 are involved in the pathogenesis of kidney injury mediated by PQ administration^38^. In our studies, we found that PQ treatment induced MMP2, MMP3 and pro-MMP9 activation, which may be regulated by IL-1β, TNF-α, IFN-γ.

In conclusion, PQ treatment targeted and damaged podocytes, leading to glomerular injury. Podocyte damage is considered not only a cause of proteinuria, but also a critical step in the progression of various glomerular diseases. Recent studies indicated that podocytes may aggravate glomerular injury by their capacity to modulate inflammation and produce ROS. Podocytes express the chemokine receptor Toll-like receptor 4, which is up-regulated in glomerulonephritis, where it may mediate glomerular injury by modulating chemokine expression and recruitment of inflammatory cells^39^,^40^. Increased ROS generation by podocytes is implicated in the progression of various glomerular diseases such as type I diabetes and puromycin nephrosis. Severe inflammation and oxidative stress are the prime factors in the pathogenesis of kidney injury and high mortality in PQ poisoning. Therefore, to further elucidate the critical role of podocytes would provide new insights into PQ poisoning therapy. Triple BMCs co-treatments attenuated kidney injury, improved renal function, and reduced mortality in PQ-induced AKI. In particular, the anti-inflammatory effects of BMCs on cytokine and chemokine production were likely responsible for the amelioration of kidney injury following PQ administration. Additionally, we found that a single dose of PQ (55 mg/kg BW) administration induced central airways resistance and pulmonary parenchyma resistance elevation and compliance reduction with most significant changes in day 3, in our unpublished data. Triple BMCs co-treatment restored the respiratory function, which also related to the survival benefits. Our study, however, showed that the anti-inflammatory effect of exogenous BMCs was closely related to the frequency of administration. This present study provides substantial evidence for the development of BMCs transplantation therapy for AKI patients, although an optimal BMCs transplantation protocol, long-term safety data, and further analysis of changes to the kidney microenvironment deserve additional investigation.

## Materials and Methods

### Animal experimental protocol and survival analysis

All animal procedures were reviewed and approved by the Animal Care Committee of the National Taiwan University, College of Medicine (IACUC’s number: 20120432), and the Committee recognizes that the proposed animal experiment follows the Animal Protection Law by the Council of Agriculture, Executive Yuan, R.O.C. and the guideline as shown in the Guide for the Care and Use of Laboratory Animals as promulgated by the Institute of Laboratory Animal Resources, National Research Council, USA. Female C57BL/6 mice aged eight weeks were obtained from the National Taiwan University, College of Medicine, Laboratory Animal Center (Taipei, Taiwan). The animals were randomly divided into experimental groups, and the five groups were treated as follows: (1) the control group (n = 15) was administered 10 ml/kg saline; (2) the PQ-only group (n = 44) was administered PQ (55 mg/kg BW) by ip injection; (3) PQ + BMCs (1) group (n = 10) was treated with single injection of BMCs (1.8 × 10^6^ cells) intravenously at 3 hours after PQ administration; (4) the PQ + BMCs (3) group (n = 15) was treated with triple injection of BMCs (5.4 × 10^6^ cells) intravenously at 3, 24 and 48 hours after PQ administration; and (5) the PQ + DEX group (n = 9) was ip injected with 10 mg/kg of DEX at 3 hours after PQ administration. The survival rate was recorded at days 1–6. The mice were sacrificed on days 1, 2, 3 and 6 after PQ exposure to evaluate the therapeutic efficiency of BMCs and DEX.

### BMCs isolation

According recently studies and our unpublished data, VEGF pre-stimulated mesenchymal stem cells (MSCs) existed a better migration capacity, which promoted us to choose the VEGF pre-stimulated BMCs in PQ induced acute kidney injury^41^,^42^,^43^. Ten-week-old C57BL/6 male mice were treated with recombinant mouse vascular endothelial growth factor (VEGF) (80 ng in 100 μl PBS; ip) once per day for 5 days. BMCs were harvested on day 6, the tibias and femurs were flushed three times with ice-cold PBS, and the samples were pooled. The pooled samples were centrifuged at 1,300 g for 5 min, re-suspended in PBS, sieved through a 70-μm mesh, re-suspended in PBS at 1.8 × 10^8 ^cells/ml, and kept on ice until use.

The surface marker expression of BMCs was assessed by flow cytometry (Beckman Coulter). BMSCs were suspended (1 × 10^6 ^cells/ml) and stained with Allophycocyanin (APC)-conjugated CD105 (Biolegend), APC-conjugated CD90 (Biolegend), R-phycoerythrin (PE)-conjugated CD29 (Biolegend), APC-conjugated CD31 (Biolegend), PE-conjugated CD34 (Biolegend), or PE-conjugated CD45 (Biolegend). After 24 hours adherent culture, the morphology of BMCs were recorded by microscope.

To trace the BMCs *in vivo*, the BMCs were labeled with the lipophilic fluorescent PKH dye (PKH26, Sigma-Aldrich) according to manufacturer’s instructions, and 96.53 ± 0.91% of BMCs labeled with PKH26 demonstrated evidence of labeling by flow cytometry (Supplementary Fig. S2A). PKH 26 fluorescence detection was performed on frozen sections of kidney on day 3 in PQ + BMCs (3) mice.

### Assessment of renal function

Blood and urine samples were collected from the control group, PQ-only group and the triple BMCs co-treatment group on days 1, 2, 3 and 6. Mouse orbital venous blood was obtained using a glass capillary. The urine of each mouse was collected from a metabolic cage on the same day. Serum was used to analyze BUN and CRE. Urine was obtained to analyze urinary total protein and CRE using a Hitachi 7070 automatic biochemical analyzer (Hitachi, Tokyo, Japan).

### Histopathological examination

Kidney tissue was excised and fixed in 10% formaldehyde for routine histology techniques. These tissues were dehydrated in an ascending grade of ethanol, cleared in xylene and embedded in paraffin. The paraffin–embedded sample was cut into 3-μm sections for histopathological examination. Tissue sections were de-paraffinized in xylene and stained with hematoxylin and eosin (HE) (Muto Pure Chemicals, 3204–2). Histopathological examinations of the kidneys were performed by an independent pathologist and one of the investigators.

### Immunofluorescence staining

Tissue sections (3 μm) were de-paraffinized in xylene and re-hydrated utilizing standard procedures. Antigen retrieval was conducted with citrate buffer (0.01 M, pH 6.0) through heating incubation, and treatment with 3% hydrogen peroxide removed endogenous peroxidase activity. Tissue sections were subjected to blocking in 3% bovine serum albumin (BSA) followed by incubation with the primary antibody overnight. Double immunofluorescence staining was performed using anti-WT-1 antibody (Santa Cruz, sc-14026; 1:500) as a nuclear marker of podocytes and anti-synaptopodin antibody (Santa Cruz, sc-21537; 1:200) as a marker of podocytes. Neutrophil infiltration of the kidney was assessed by anti-Ly6G antibody (1:1,000) identification. After washing, tissue sections were treated with the corresponding secondary antibody and prepared with a relative secondary antibody control. Finally, tissue sections were subjected to DAPI nuclear staining. Photographs were taken with inverted fluorescence microscope (ZEISS, Axiovert 200 M Inverted Microscope), and images were acquired using TissueQuest software (TissueGnostics, Vienna, Austria). Quantification of immunofluorescence was performed using TissueGnostics microscopy scanning and HistoQuest software analysis (TissueGnostics, Vienna, Austria). Quantification of glomerulus damage was performed by analyzing synaptopodin and WT-1 distribution ratios in the glomerulus. One hundred glomeruli were evaluated in each experimental group. Neutrophil infiltration of the kidney was observed by staining with Ly6G, a neutrophil marker. Ly6G-positive cell ratios were measured in five mice from each group.

### Urinary podocin level

The urinary podocin level was determined using commercial mouse podocin enzyme-linked immunosorbent assay (ELISA) kits (MyBioSource, MBS722280). The assay procedure was performed according to the manufacturer’s instructions. Urine samples were collected from the control group, PQ-only group and the triple BMCs co-treatment group on days 1, 2, 3 and 6. The urinary podocin content was normalized to the urinary CRE concentration. All samples were assayed in triplicate.

### Cytokine level

The cytokine content of the kidneys was measured with a mouse cytokine array that included 97 cytokines (RayBiotech, Norcross, GA, AAM-CYT-3 and AAM-CYT-4). Kidney samples were collected from the control group, PQ-treated group and triple BMCs co-treatment group on days 1, 2, 3 and 6. Kidney supernatant was obtained as follows. First, the kidneys were removed, minced, and homogenized in lysis buffer. The homogenate was then centrifuged at 9,000 × g for 30 min at 4 °C, the pellet was discarded, and the supernatant was stored at –70 °C until use. The cytokine content of the kidney supernatant was performed according to the manufacturer’s instructions. Briefly, the samples were incubated with the membranes for 2 h at room temperature. After washing, the membranes were incubated with biotin-conjugated anti-cytokine primary antibodies, followed by washes and development with HRP-conjugated streptavidin. The membrane was exposed to X-ray film for 30–60 s. The intensities of the cytokine signals were quantified using Image J software (NIH, Bethesda). The densities of the cytokine spots were normalized according to the blank control spot, and a positive spot was used as the internal control to measure the cytokine content of individual samples.

### Cell culture and cellular viability assay

Renal cell lines used in this study included murine podocyte (PDCs), murine glomerular mesangial cells (GMCs, MES-13), rat proximal tubular epithelial cells (PTECs, NRK-52E), and murine distal convoluted tubular epithelial cells (DCTCs). PDCs from a conditionally immortalized cell line were cultured, as described previously^44^. Cells were cultured under growth-permissive conditions at 33 °C in RPMI-1640 medium (Sigma) supplemented with 10% FBS (GIBCO) and 20 U/ml mouse recombinant interferon-gamma (IFN-γ, Sigma). To induce differentiation, podocytes were maintained in non-permissive conditions at 37 °C in the absence of IFN-γ for at least 2 weeks and were used for experiments. GMCs (MES-13) were cultured in DMEM medium (Sigma) supplemented with 10% FBS. PTECs (NRK-52E cells) were cultured in DMEM medium containing 5% FBS and 2 mM glutamine (Invitrogen). DCTCs were maintained in DMEM low glucose medium (Sigma) with 5% FBS. After serum starvation for 16 h, cells were treated with PQ for a range, as indicated. After 24 h, WST-1 assay (Roche) was performed to determine cell viability according to the manufacturer’s instructions. Cell viability was expressed as a percentage of the non-treated group, and the EC_50_ values were determined.

### Statistical analysis

Each experiment was repeated at least three times, and the results are presented as the mean ± SE. Statistically significant differences between groups were determined using Student’s *t* test. *P* values less than 0.05 were considered significant.

## Additional Information

**How to cite this article**: Gu, S.-Y. *et al.* Unfractionated bone marrow cells attenuate paraquat-induced glomerular injury and acute renal failure by modulating the inflammatory response. *Sci. Rep.* **6**, 23287; doi: 10.1038/srep23287 (2016).

## Supplementary Material

Supplementary Information

## Figures and Tables

**Table 1:** The BUN, CRE and total protein levels in serum and urine of the control group, PQ-only group and triple BMCs co-treatment with PQ group.

	Serum BUN (mg/dl)			Serum CRE (mg/dl)			uPCR		
	CTR	PQ	PQ + BMCs (3)	CTR	PQ	PQ + BMCs (3)	CTR	PQ	PQ + BMCs (3)
**Day 1**	27.26 ± 1.39	145.81 ± 8.66**	54.21 ± 22.71##	0.29 ± 0.09	0.84 ± 0.47**	0.37 ± 0.03**#	3.94 ± 1.83	15.18 ± 4.15**	6.19 ± 1.31*##
**Day 2**	25.80 ± 1.49	114.38 ± 23.07**	52.11 ± 25.11##	0.20 ± 0.05	0.71 ± 0.57**	0.43 ± 0.03##	3.27 ± 2.07	10.78 ± 5.45**	7.54 ± 1.36*##
**Day 3**	27.73 ± 1.52	107.78 ± 25.10**	40.47 ± 38.40**##	0.24 ± 0.05	0.73 ± 0.61**	0.47 ± 0.09**	2.78 ± 2.09	6.63 ± 3.78**	4.56 ± 1.21*##
**Day 6**	19.93 ± 1.24	55.02 ± 5.00**	34.74 ± 2.51**##	0.20 ± 0.01	0.63 ± 0.40**	0.28 ± 0.02**##	3.52 ± 2.38	6.53 ± 3.36	4.35 ± 0.68*##

Data are expressed as the mean ± SE n = 6. uPCR, urine protein and CRE ratio.

^*^p < 0.05 relative to control group; **p < 0.01 relative to control group.

^#^*p* < 0.05 relative to PQ group; ^##^*p* < 0.01 relative to PQ group.

CTR: control group; PQ: PQ-only group; PQ + BMCs (3): triple BMCs co-treatment with PQ groups.

**Table 2:** Quantification of Ly6G expression in kidneys of the control group, PQ-only group and triple BMCs co-treatment with PQ group.

	Neutrophil/mm^2^		
	CTR	PQ	PQ + BMCs (3)
**Day 1**	0.83 ± 0.03	1.97 ± 0.18**	1.63 ± 0.25*
**Day 2**	0.84 ± 0.02	3.00 ± 0.15**	1.17 ± 0.03**
**Day 3**	0.84 ± 0.03	4.82 ± 0.28**	1.17 ± 0.02**##
**Day 6**	0.83 ± 0.02	3.83 ± 0.11**	1.10 ± 0.03**##

Data are expressed as the mean ± SE n = 6.

^*^p < 0.05 relative to control group; ^**^p < 0.01 relative to control group.

^#^*p* < 0.05 relative to PQ group; ^##^*p* < 0.01 relative to PQ group.

CTR: control group; PQ: PQ-only group; PQ + BMCs (3): triple BMCs co-treatment with PQ group.
