# Supplementary material for: Unfractionated bone marrow cells attenuate paraquat-induced glomerular injury and acute renal failure by modulating the inflammatory response
Source: Sci Rep. 2016 Mar 18;6:23287. doi: 10.1038/srep23287 (PMC4796871; doi:10.1038/srep23287)
Supplement: Supplementary Information [file srep23287-s1.pdf]

Unfractionated bone marrow cells attenuate paraquat-induced glomerular injury and acute renal failure by modulating the inflammatory response

Sing-Yi Gu, Ti-Yen Yeh, Shih-Yi Lin and Fu-Chuo Peng*

(Supplementary Information)

Supplementary Figure S1.

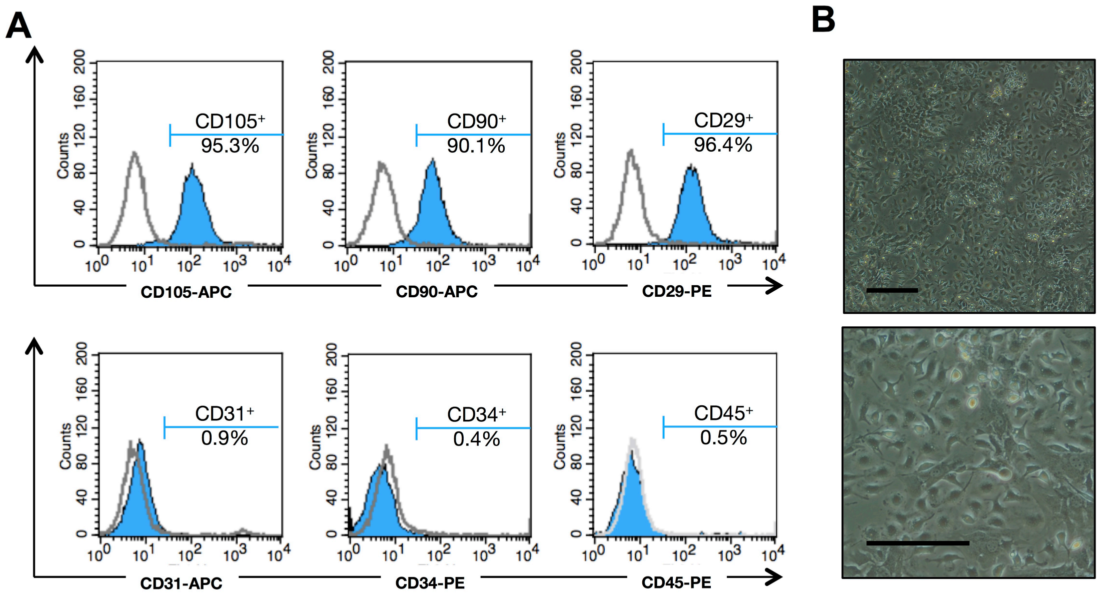

Supplementary Figure S1. BMCs identification. (A) Using flow cytometry analysis, 95.3%, 90.1%, and 96.4% of the BMCs were positive for mesenchymal lineages markers, CD105, CD90, and CD29, respectively. Hematopoietic lineages markers, CD31, CD34, and CD45 positive only were account for ≤2% of the BMCs population. (B) After 24 h of adherent growth, the BMCs morphologically resembled fibroblasts by microscope recording.

Supplementary Figure S2.

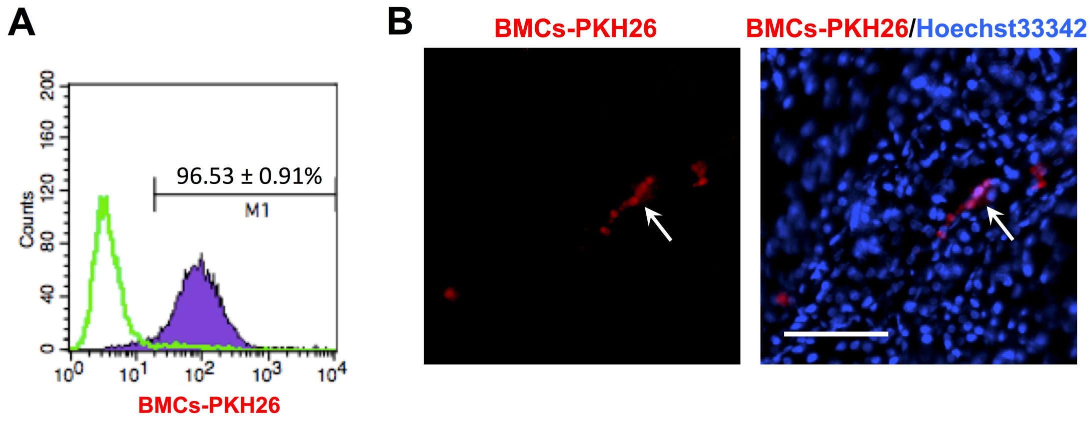

Supplementary Figure S2. Labeling of BMCs for *in vivo* tracking. (A) PKH26 labeling of BMCs. Flow cytometry analysis showed that 96.53 ± 0.91% of BMCs were labeled with PKH26. (B) PKH 26 fluorescence were detected on day 3 in PQ+BMCs(3) mice, as indicated by arrows. Hoechst 33342 (2 μM) was used as a nuclear stain. Scale bar is 100 μm.
